# Effectiveness of a Randomized Controlled Trial of Individual Reminiscence Therapy on Cognition, Mood and Quality of Life in Azorean Older Adults with Neurocognitive Disorders

**DOI:** 10.3390/jcm10225395

**Published:** 2021-11-19

**Authors:** Susana I. Justo-Henriques, Enrique Pérez-Sáez, João L. Alves Apóstolo, Janessa O. Carvalho

**Affiliations:** 1 Health Sciences Research Unit: Nursing (UICISA: E), Nursing School of Coimbra (ESEnfC), 3004-011 Coimbra, Portugal; apostolo@esenfc.pt; 2 National Reference Centre for Alzheimer’s and Dementia Care, Imserso, 37008 Salamanca, Spain; enriqueperezsaez@imserso.es; 3 Psychology Department, Bridgewater State University, Bridgewater, MA 02325, USA; janessa.carvalho@bridgew.edu

**Keywords:** aging, cognitive impairment, COVID-19, dementia, executive functions, memory, mood, neurocognitive disorders, quality of life, reminiscence therapy

## Abstract

Reminiscence therapy (RT) is a form of cognitive stimulation therapy that incorporates discussion of past activities, events, and experiences to stimulate individual memories; it has had some success in treating persons with neurocognitive disorders. This research aims to evaluate the ability of individual RT, using a simple reminiscence format, to improve the overall cognitive function, memory, executive functions, emotional status, and quality of life in older adults with neurocognitive disorders who received social care and support services. A multicenter randomized controlled trial was completed in the Azores archipelago (an independent region of Portugal) using repeated measures (pre-intervention, post-intervention, and follow-up). The intervention group underwent individual RT sessions, twice weekly for 13 weeks, while the control group completed regular activities administered as part of their program. Results did not reveal any significant differences between the intervention and control groups. While results did not reveal significant effects, a number of historical and contextual factors are considered as possible explanations for the lack of effects—namely, data collection occurring during the COVID-19 global pandemic, participant cohort effects, and therapist heterogeneity.

## 1. Introduction

Major neurocognitive disorder (mNCD), characterized by the *Diagnostic Statistical Manual of Mental Disorders*, 5th edition (DSM-5) as a cognitive decline relative to a previous level of functioning [1], is one of the main causes of disability among older adults, and its prevalence is increasing due to the aging of the population. It is estimated that globally, neurocognitive disorders affect over 45 million people, and it is expected that by the year 2050, the number of those affected worldwide would surpass 130 million [2]. The absence of an effective pharmacological treatment that halts or delays the development of the disease has raised interest in non-pharmacological therapies that also seek to improve the experiences of persons with neurocognitive disorders.

One form of non-pharmacological therapy, cognitive stimulation, seeks to provide an enriching and engaging environment for participants. In NCD samples, this therapy has demonstrated improvements in cognitive functioning, social functioning, and quality of life (QoL) [3,4]. Cognitive stimulation is a recommended therapy by the National Institute for Health and Clinical Excellence for NCD patients [5].

Reminiscence therapy (RT) is a form of cognitive stimulation that incorporates discussion of past activities, events, and experiences, usually with the help of cues (e.g., photographs, home objects, and other familiar items from the past, music, any object or stimulus) that serve to stimulate individual memories. This method has had some success in older adults with mNCDs (mixed dementia samples) given relatively preserved remote autobiographical memories in this group [6,7]. The goal of this strategy is to enable the individual to connect with their past and recover a sense of personal identity [8].

A Cochrane review described positive, albeit small, effects of RT on cognition, QoL, communication, and possibly on the mood of persons with dementia [9]. Despite distinctions between two different approaches to RT (general reminiscence versus life history), the therapy modality does not seem to be as important as the format (individual or group) and the residential setting (community versus congregate living). In particular, according to the review, RT provided a small benefit on cognitive function immediately after the intervention, although post-intervention follow-up revealed no significant effects. Individual RT was slightly superior in its effects on cognition both immediately and after a follow-up period. An abridged Cochrane review also found similar modest but notably important effects of RT, with improvements in cognition in mood in individual format and communication in a group format [8]. Notably, one of the suggestions made by the authors in this review was to increase the dissemination of standardized RT protocols given the individualized and varied nature of RT.

A standardized individual RT protocol for Portuguese-speaking patients with NCD was created, piloted, and implemented, which is detailed elsewhere [10]. Exploration of a 13-week, twice weekly, deployment of that protocol in NCD patients revealed significant intervention effects for cognition and QoL but not depressive symptoms [11,12]. Further exploration of this protocol reviewed the characteristics of those who responded to the intervention, revealing those with worse baseline depressed mood, executive functioning, and QoL scores all were more likely to respond positively to this intervention [13].

The current study evaluated the effectiveness of individual RT (iRT) on cognitive functioning (global cognition, memory, and executive functions), emotional state (depression and anxiety), and QoL in older adults with mNCD. Building on the previous studies using the current iRT protocol, the current study expands in multiple ways. First, participants were from a day program in the Azores Autonomous Region, an archipelago of Portugal that is one of the four Portuguese NUTS II regions that are considered less developed [14]. We also explored self-reported anxiety. Finally, we explored post-intervention effects in a three-month follow-up.

## 2. Method

This study analyzed a clinical trial of iRT for people with NCD (clinicaltrials.gov ID: NCT04658394), which was designed as a multicenter, single-blind, randomized, parallel two-arm (iRT vs. regular activities, 1:1 ratio), controlled trial. Participants in the intervention group received two 50 min weekly sessions of iRT for 13 weeks in addition to their regular daily activities. Participants in the control group underwent only their regular activities. Participants were assessed at baseline (T0), after the iRT intervention (T1), and at a 3-month follow-up (T2). Participants understood that participation in the study was voluntary.

### 2.1. Participants

Recruitment took place from 15 to 19 February 2021. Recruitment included contacting social care institutions in the Azores, through the supervising government entity, to participate in an RCT on iRT for people with NCD; overall, 14 care institutions showed interest in the study and contacted the research team, and 12 institutions agreed to collaborate. The institutions were then asked to select participants they believed could participate in the study based on the inclusion criteria, resulting in 170 participants completing the eligibility assessment. After baseline assessment, 122 participants were selected from 12 institutions: 62 (50.8%) allocated to the iRT group and 60 (49.2%) to the control group.

Inclusion criteria included a diagnosis of neurocognitive disorder (major or minor) from their general practitioner based on DSM-5 criteria; reviewed and signed consent form; intact language expression and comprehension; access to autobiographical information about the participants through family members or caregivers (via the socio-family questionnaire as part of the protocol); aged 65 years or older; being a native Portuguese speaker; regularly attending an institution that provides social care and support services for older adults.

Exclusion criteria included an acute or severe illness that prevented participation in the intervention sessions; severe sensory and physical limitations; severe disconnection from the environment and minimal attention span; the presence of severe neuropsychiatric symptoms or uncontrolled delirium that prevented participation in the sessions; traumatic life history or experienced adverse events that discouraged participation; history of adverse reactions during iRT sessions or similar activities; severe or total functional dependence (indicated by a score of 13 points or lower in a modified version of the Barthel Index, with scores ranging from 0 to 20 [15,16]).

Participants who met the inclusion criteria were enrolled, and baseline assessments were completed. Participants were then randomly allocated to either the control group or the experimental group. Due to the multicenter nature of the study, each institution had two groups, the intervention and control groups, with a 1:1 ratio. A non-stratified permuted block randomization process (with variable block size) was carried out using the software DatInf^®^ RandList (version 1.5, DatInf GmbH, Tübingen, Germany) by one of the study principal investigators blinded to baseline scores and demographics of the participants. The overall number of participants in each institution ranged from two to eight persons. Groups allocation was unknown to participants, therapists, or institution staff until the intervention started. Evaluators remained blinded to participant allocation until endpoint assessments were completed. Enrollment of participants was performed by the researchers responsible for communicating with the institutions who assessed eligibility. The therapists at each institution administered the intervention.

### 2.2. Intervention

As noted, the iRT protocol used in this study has been described elsewhere, including specific information on activities and materials for each session [10]. It includes activities with cues (such as cards with images) divided into nine RT topics (means of transportation, appliances, housing, media, professions, clothing, actors and presenters, politics, and regional/local references). The cards in the regional/local references were modified based on the region where each institution was located by the institution’s therapist. Other materials included music, riddles, and themed worksheets [10]. Each iRT session lasted 50 min and had the following structure: introduction and orientation to time and place (day, month, year, season, weather, and name and address of the institution; 7 min); main RT activity (40 min); wrap up and scheduling the next session (3 min). There were no reported side effects or unintended consequences in either group.

All iRT sessions were conducted by 14 therapists (psychologists, occupational therapists, or gerontologists) who completed a 6 h training on the protocol. Principles of the therapy were discussed by two of the principal investigators, and the materials, objectives, agenda, and activities of each session were provided and reviewed.

The sessions were held at each institution. Each therapist scheduled the individual sessions, preferably in a room with minimal distractions. Each therapist always treated the same patients (i.e., there was no rotating between therapists and participants). The intervention lasted 13 weeks, from 1 March 2021 to 28 May 2021. The follow-up period ended on 20 August 2021.

The participants in the control group did not receive the iRT intervention but participated in their usual activities provided by the institution. This treatment, although varied according to the staffing available in each institution, included social interaction activities, cognitive stimulation activities, stimulation of personal skills, and administration of any prescribed dementia medication.

### 2.3. Instruments

The protocol included several tools and was administered to all participants (intervention and control groups) by trained evaluators who were blinded to the participant group. Data were collected at baseline (T0), 15 weeks post-baseline (endpoint assessment T1), and 3 months post-intervention completion (T2). The outcome measures addressed the global cognitive function, other specific cognitive functions (memory and executive function), mood (depression and anxiety), functional abilities (only used to assess for eligibility), and QoL.

The Mini-Mental State Examination (MMSE; Cronbach’s alpha = 0.89) assessed global cognitive function. Scores range from 0 to 30, with higher scores indicating better cognitive functioning [17,18,19].

The Memory Alteration Test (MAT; Cronbach’s alpha = 0.93) assessed memory function. It is an easy and quick instrument that assesses five memory domains: temporal orientation, encoding, semantic memory, free recall, and cued recall. Total scores range from 0 to 50, with higher scores indicating better memory [20,21].

The Frontal Assessment Battery (FAB; Cronbach’s alpha = 0.83) assessed executive function in several subtests: conceptualization, mental flexibility, motor programming, sensitivity to interference, inhibitory control, and environmental autonomy. Scores range from 0 to 18, with higher scores indicating better executive functioning [22,23].

The Geriatric Depression Scale-15 (GDS-15; Cronbach’s alpha = 0.83) measured depressive symptoms. It is considered a reliable tool to screen depressive symptoms in older adults, in a dichotomous format (yes/no answers). Scores range from 0 to 15, with higher scores indicating more severe depressive symptoms [24,25,26].

The Geriatric Anxiety Inventory (GAI) (Cronbach’s alpha = 0.964) measured anxiety symptoms. It is a self-report measure considered a reliable tool to screen for symptoms of anxiety in older adults. Scores range from 0 to 20 with higher scores indicating more severe anxiety symptoms [27,28].

The Quality of Life in Alzheimer’s Disease scale (QoL-AD; Cronbach’s alpha = 0.87) evaluated QoL. This 13-item scale assesses the QoL in people diagnosed with dementia, gathering information from the patient about the following domains: perceived health, mood, physical condition, interpersonal relationships, hobbies, decision-making skills, and life as a whole. Scores range from 13 to 52, with higher scores indicating better QoL [29,30].

### 2.4. Data Analysis

Chi-square tests for categorical variables and *t*-tests for continuous variables were performed to determine whether the groups were homogenous prior to treatment. No imputation of missed data was made; thus, only data from participants who completed the follow-up assessment were analyzed.

The effects of iRT on outcomes (MMSE, FAB, MAT, GDS-15, GAI, QOL-AD) were analyzed using 2 × 3 repeated-measures mixed ANOVAs, with group assignment as a between-subjects factor (iRT, control) and time as a within-subjects factor (baseline T0, endpoint T1, follow-up T2). The main effects of interest were the group × time interactions. The Greenhouse–Geisser correction was used when the sphericity test was significant. The level of significance was set at *p* < 0.05 for all analyses. Statistical analysis was performed using IBM SPSS Statistics (version 20, IBM Corp, Armonk, NY, USA).

## 3. Results

### 3.1. Sociodemographic and Clinical Characteristics

Table 1 shows the participants’ characteristics and assessment scores at baseline. Although our inclusion criteria referred to both major and minor NCD, the final sample consisted only of people with major NCD. No significant differences were found between the intervention and control groups regarding age, gender, clinical diagnosis, educational level, marital status, type of institution attended, and immigrant family (Table 1). No significant differences were found between the intervention and control groups regarding clinical condition or baseline mean scores for MMSE, FAB, TAM, GDS-15, GAI, and QoL-AD.

**Figure 1:**
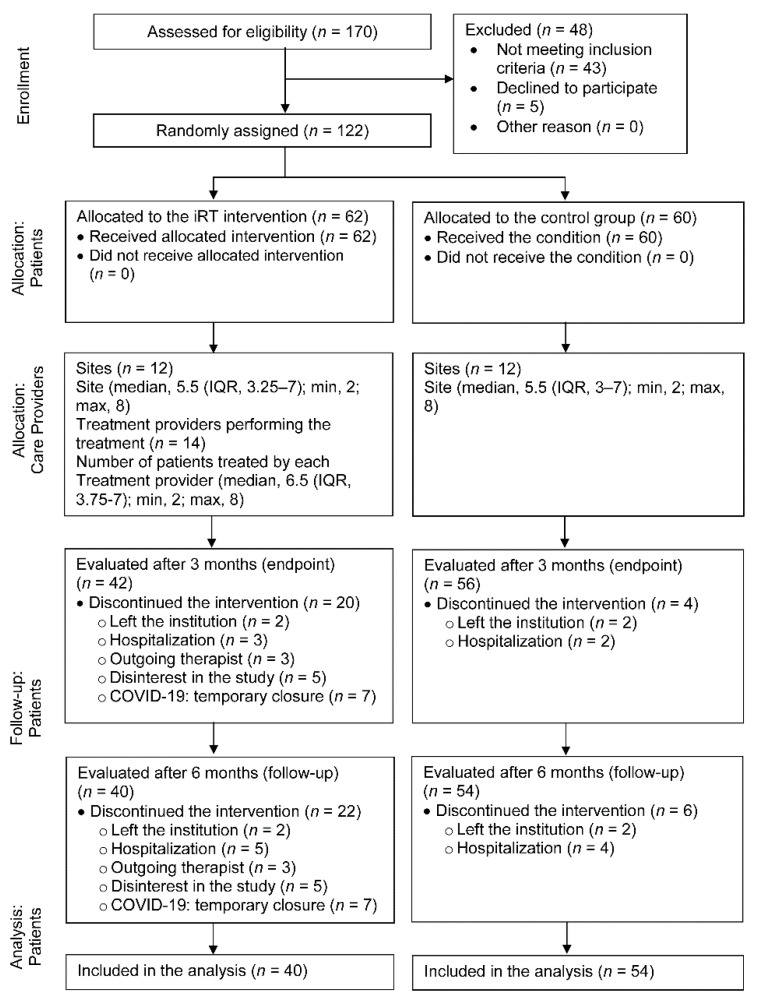
CONSORT diagram of participant flow through the study. Abbreviations: IQR = interquartile range; iRT = individual reminiscence therapy.

Of the 62 participants in the iRT group, 40 (64.5%) completed the three assessments. The intervention could not be completed for 20 participants (32.3%) who were not assessed at T1; two other participants (3.2%) failed to complete follow-up and were not assessed at T2 (see Figure 1). These participants withdrew from the study for a variety of reasons: two left the institution where the sessions occurred; five were not interested in continuing in the study; five were hospitalized; seven discontinued the intervention due to the temporary closure of the daycare center as a consequence of COVID-19 pandemic, and three due to departure of the therapist from the social care institution. No serious adverse events related to the trial were recorded.

Of the 60 participants in the control group, 4 (6.7%) did not complete the endpoint assessment and 2 others (3.3%) could not be assessed at follow-up, resulting in a total of 54 participants (90%) completing the three assessments. The reasons for withdrawal included two leaving the institution where the study was conducted and four being hospitalized.

For those participants who completed the follow-up assessment, no significant differences were found between the intervention and control groups regarding demographic variables, clinical condition, or baseline outcome mean scores.

### 3.2. Effects of iRT

#### 3.2.1. MMSE

The 2 × 2 ANOVA (Table 2) did not show a significant group × time interaction, *F*(1.740, 160.09) = 2.158, *p* = 0.126, η_p_^2^ = 0.023.

#### 3.2.2. FAB

ANOVA for FAB scores (Table 2) did not show a significant group × time interaction, *F*(2, 184) = 1.398, *p* = 0.250, η_p_^2^ = 0.015.

#### 3.2.3. MAT

The ANOVA for MAT (Table 2) did not show a significant group × time interaction, *F*(1.785, 164.197) = 0.361, *p* = 0.673, η_p_^2^ = 0.004.

#### 3.2.4. GDS-15

ANOVA for GDS-15 (Table 2) did not show a significant group × time interaction, *F*(1.861, 171.217) = 0.013, *p* = 0.983, η_p_^2^ = 0.000, since both iRT and control groups significantly improved their scores at T1.

#### 3.2.5. GAI

The ANOVA for GAI (Table 2) did not show a significant group × time interaction, *F*(2, 184) = 0.067, *p* = 0.936, η_p_^2^ = 0.001, since both groups reduced their scores through the trial.

#### 3.2.6. QoL-AD

No significant Group × Time interaction, *F*(2, 184) = 0.044, *p* = 0.957, η_p_^2^ = 0.000 was found in the ANOVA for QoL-AD (Table 2).

### 3.3. Adherence to Intervention

The adherence to iRT sessions was medium (Table 3). The mean attendance of participants was 19.7 sessions (out of 26 sessions). It should be noted that 69.4% of participants attended more than 20 sessions; specifically, 41.9% attended all sessions, and 50.0% attended 25 or 26 sessions.

The reasons mentioned for not attending the sessions include leaving the institution, disinterest in the study, or COVID-19-related factors such as temporary closure, unavailability of the therapist to attend the sessions, and hospitalization.

### 3.4. Degree of Participation during the Intervention

After analyzing the individual records of each session, we were able to obtain data regarding the level of collaboration of participants throughout the intervention program, operationalized by active participation in the RT activities; participation was high. Of the 1221 iRT sessions carried out, participants collaborated in 1039 (85.1%) of them. Only in 164 (13.4%) sessions did participants appear passive, and only in 18 (1.5%) did participants not cooperate, showing drowsiness or a very low level of attention and concentration that prevented an adequate performance in the proposed activities.

## 4. Discussion and Conclusions

This study presents the results of an RCT on the immediate and 3-month follow-up effects of a 13-week iRT intervention for people with mNCDs. The results did not show a significant effect of the iRT intervention on global cognition (assessed through MMSE), though the iRT group improved their scores upon completion of the intervention. No significant effects for the intervention were found on any of the other outcomes, including memory, executive functions, mood (anxiety and depressive symptoms), or QoL. This is an unexpected result, as the current iRT protocol has shown positive effects on global cognition, memory, and QoL in other Portuguese-speaking populations with NCD [11,12].

Although there was moderate adherence to the intervention (with 41.9% of participants receiving all sessions) and a high degree of participation in the iRT sessions (participants collaborated in 85.1% of sessions), the number of dropouts was high (32.2%). Some studies that found no significant effects of iRT had a dropout rate of over 40% and low adherence [31].

There are a number of other factors that could explain our lack of significant treatment effects. First, the intervention and data collection occurred during the COVID-19 global pandemic, which is likely to have affected mood and cognitive functioning given associated health concerns and isolation, particularly on an island with travel mostly occurring by air (and imposed restrictions). During the period of the COVID-19 global pandemic, in the initial phase, participants saw confinement and isolation measures, while in the final phase, restrictions were somewhat eased, including restarting family visits. Finally, cohort effects might have played a role. The current sample experienced a political revolution in the 1970s in which a military coup overthrew an authoritarian regime, and the Azores islands were granted autonomy by the Portuguese government; thus, cultural and personal past events may not have been experienced similarly in the current sample relative to the past participants who had positive effects from the iRT, despite making some prior modifications to the content of the intervention (photos) to mitigate past experience differences. Lastly, as previously stated, the effectiveness of reminiscence therapy has been mixed [9], and thus, this minimal effect is not entirely surprising.

The study had some limitations that must be considered in the interpretation of its results and implications. The medium size of the sample and relatively short intervention duration may have limited our capacity to detect cognitive or behavioral changes that could have emerged with a longer intervention. Additionally, we had limited information on the participants’ medications or other treatments received. Regarding diagnosis, the lack of confirmation through biomarkers makes it possible that those with other amnestic syndromes (e.g., Limbic-predominant age-related TDP-43 encephalopathy [32], were included because they were misclassified as probable Alzheimer’s disease. Regarding outcomes, more comprehensive cognitive measures (e.g., ADAS-Cog) would provide more information on cognitive changes, though we were limited to cognitive screening measures for the current study. Along these lines, our mNCD sample was diagnostically heterogeneous, which makes it difficult to conclude specific intervention effects in a diagnostic group. We also had a heterogeneous group of therapists and evaluators with various professional skills and experiences, though notably, their training was standardized. As for the therapists, they were psychologists, occupational therapists, and gerontologists. In the protocol training, we found that some therapists had no previous experience with cognitive stimulation programs focused on reminiscence therapy; thus, they may have encountered difficulties in correctly applying the intervention principles and addressing the standardized protocol. Thus, it is difficult to generalize the results of this trial to other demographic and clinical groups.

Overall, the current iRT intervention findings provide important contributions to the literature on this topic. While our results revealed no significant effects, it adds to our understanding and exploration of the current iRT protocol [10] that will help the authors determine whether modifications to the intervention or outcome variables should be considered. Additionally, future research should include detailed characterization of the profiles of the professionals involved—namely, educational level, profession, years of professional experience, years of experience with therapy, hours of complementary training, and relevant to the area of study acquired in the last five years, type of working relationship and years of service, age, and gender.

## Figures and Tables

**Table 1:** Sociodemographic and clinical characteristics of the sample. Results of between-group comparisons at baseline.

	Overall Sample (*n*= 122)	iRT Group (*n*= 62)	Control Group (*n*= 60)		
Age				*t*	*p* value
Mean (SD)	80.22 (7.03)	80.82 (6.69)	79.6 (7.37)	0.960	0.339
Range	65–94	67–93	65–94		
Gender				*x* ^2^	*p* value
Male	32 (26.2%)	17 (27.4%)	15 (25.0%)	0.092	0.761
Female	90 (73.8%)	45 (72.6%)	45 (75.0%)		
Educational level					
No literacy	21 (17.2%)	13 (21.0%)	8 (13.3%)	6.364	0.272
1 to 2 years	6 (4.9%)	1 (1.6%)	5 (8.3%)		
3 to 4 years	79 (64.8%)	38 (61.3%)	41 (68.3%)		
5 to 6 years	11 (9.0%)	6 (9.7%)	5 (8.3%)		
7 to 11 years	2 (1.6%)	2 (3.2%)	0 (0.0%)		
Over 11 years	3 (2.5%)	2 (3.2%)	1 (1.7%)		
Marital status					
With partner	16 (13.1%)	9 (14.5%)	7 (11.7%)	0.217	0.641
Without partner	106 (86.9%)	53 (85.5%)	53 (88.3%)		
Type of institution attended					
Long-term care center	99 (81.1%)	51 (82.3%)	48 (80.0%)	0.102	0.750
Day center	23 (18.9%)	11 (17.7%)	12 (20.0%)		
Immigrant family					
Yes	45 (36.9%)	23 (37.1%)	22 (36.7%)	0.002	0.961
No	77 (63.1%)	39 (62.9%)	38 (63.3%)		
Clinical diagnosis					
Alzheimer’s disease	72 (59.0%)	42 (67.7%)	30 (50.0%)	4.621	0.328
Vascular dementia	25 (20.5%)	9 (14.5%)	16 (26.7%)		
Frontotemporal degeneration	13 (10.7%)	5 (8.1%)	8 (13.3%)		
Parkinson’s disease	10 (8.2%)	5 (8.1%)	5 (8.3%)		
Traumatic brain injury	2 (1.6%)	1 (1.6%)	1 (1.7%)		
Baseline assessment	Mean (SD)	Mean (SD)	Mean (SD)	*t*	*p* value
MMSE	21.75 (3.17)	22 (3.52)	21.48 (2.78)	0.898	0.371
Range	12–27	12–27	16–27		
FAB	9.23 (3.33)	8.97 (3.16)	9.5 (3.5)	−0.883	0.379
Range	0–18	0–15	4–18		
MAT	25.15 (8.89)	24.15 (9.37)	26.18 (8.32)	−1.269	0.207
Range	4–45	4–42	7–45		
GDS−15	7.05 (3.35)	6.65 (3.16)	7.47 (3.51)	−1.360	0.176
Range	0–15	0–14	1–15		
GAI	11.73 (5.94)	10.90 (5.41)	12.58 (6.37)	−1.572	0.119
Range	0–20	0–20	0–20		
QoL-AD	26.33 (5.42)	26.53 (5.15)	26.12 (5.73)	0.422	0.674
Range	16–40	17–37	16–40		

Abbreviations: FAB= Frontal Assessment Battery; GAI= Geriatric Anxiety Inventory; GDS-15= Geriatric Depression Scale-15; iRT= individual reminiscence therapy; MAT= Memory Alteration Test; MMSE= Mini-Mental State Examination; QoL-AD= Quality of Life in Alzheimer’s Disease Scale.

**Table 2:** Results of repeated-measures ANOVA.

	iRT (*n* = 40)			Control (*n* = 54)			Moment × Group			
	T0 Mean (SD)	T1 Mean (SD)	T2 Mean (SD)	T0 Mean (SD)	T1 Mean (SD)	T2 Mean (SD)	df	*F*	*p* Value	η_p_^2^
MMSE	22.63 (2.86)	23.73 (3.78)	23.05 (4.88)	21.52 (2.69)	21.69 (4.03)	22.30 (4.50)	1.740, 160.09	2.158	0.126	0.023
FAB	8.77 (2.68)	8.75 (2.84)	8.30 (2.84)	9.39 (3.38)	8.67 (3.33)	9.11 (3.23)	2, 184	1.398	0.250	0.015
MAT	25.55 (8.57)	28.30 (9.51)	29.38 (10.71)	25.72 (8.05)	28.35 (8.43)	28.54 (9.94)	1.785, 164.197	0.361	0.673	0.004
GDS-15	6.23 (3.27)	5.18 (3.49)	5.33 (3.81)	7.39 (3.66)	6.43 (3.48)	6.56 (4.03)	1.861, 171.217	0.013	0.983	0.000
GAI	10.82 (5.88)	9.67 (6.31)	8.85 (6.29)	12.44 (6.55)	11.06 (6.37)	10.11 (6.73)	2, 184	0.067	0.936	0.001
QoL-AD	26.38 (5.74)	28.02 (6.40)	27.33 (6.42)	26.30 (5.64)	27.26 (6.38)	26.54 (6.30)	2, 184	0.044	0.957	0.000

Abbreviations: FAB = Frontal Assessment Battery; GAI = Geriatric Anxiety Inventory; GDS-15 = Geriatric Depression Scale-15; iRT = individual reminiscence therapy; MAT = Memory Alteration Test; MMSE = Mini-Mental State Examination; QoL-AD = Quality of Life in Alzheimer’s Disease Scale; T0 = baseline assessment; T1 = endpoint assessment; T2 = follow-up assessment.

**Table 3:** Attendance to individual reminiscence therapy sessions.

Attendance	*n* = 62	%
Sessions attended		
M (SD)	19.69 (8.28)	
Number of sessions attended		
Between 0 and 5	4	6.4
Between 6 and 10	12	19.4
Between 11 and 15	3	4.8
Between 16 and 20	0	0
21 and over	43	69.4
21	3	4.8
22	4	6.5
23	2	3.3
24	3	4.8
25	5	8.1
26	26	41.9

## Data Availability

The data that support the findings of this study are available from the corresponding author upon reasonable request. The data are not publicly available due to privacy/ethical restrictions.
